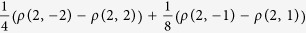# Elliptical polarization of near-resonant linearly polarized probe light in optically pumped alkali metal vapor

**DOI:** 10.1038/srep43066

**Published:** 2017-02-20

**Authors:** Yingying Li, Zhiguo Wang, Shilong Jin, Jie Yuan, Hui Luo

**Affiliations:** 1 College of Optoelectronic Science and Engineering, National University of Defense Technology, Changsha 410073, China; 2 Interdisciplinary Center for Quantum Information, National University of Defense Technology, Changsha 410073, China

## Abstract

Optically pumped alkali metal atoms currently provide a sensitive solution for magnetic microscopic measurements. As the most practicable plan, Faraday rotation of linearly polarized light is extensively used in spin polarization measurements of alkali metal atoms. In some cases, near-resonant Faraday rotation is applied to improve the sensitivity. However, the near-resonant linearly polarized probe light is elliptically polarized after passing through optically pumped alkali metal vapor. The ellipticity of transmitted near-resonant probe light is numerically calculated and experimentally measured. In addition, we also analyze the negative impact of elliptical polarization on Faraday rotation measurements. From our theoretical estimate and experimental results, the elliptical polarization forms an inevitable error in spin polarization measurements.

Optical pumping offers an efficient means for the generation of a high degree of polarized alkali metal atoms and has been used in a diverse range of areas, such as fundamental physics^1^, geophysical exploration^2^, and medical diagnoses^3^,^4^. Accurate knowledge of the spin polarization is necessary in applications of the optically pumped alkali metal atoms. For example, the detection of magnetic field with an atomic magnetometer scales with the polarization of the alkali metal atoms^5^. Among numerous techniques, Faraday rotation of linearly polarized light is predominantly used for the spin polarization measurement^6^,^7^. When the alkali metal vapor has been polarized, it induces a Faraday rotation angle *θ* on the polarization plan of linearly polarized probe beam^8^.

Consider the alkali metal atoms confined to a cell with a length *l*along the propagate direction of the probe beam. When the probe beam is detuned far from the atomic resonance (much greater than the hyperfine splitting), the Faraday rotation angle is equal to^9^


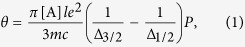


where Δ_3/2_ is the probe beam detuning from the D2 line transition and Δ_1/2_ is the probe beam detuning from the D1 line. *e* is the electron charge, [A] is the number density of the alkali-metal vapor, *m* is the electron mass, *c* is the speed of light, and *P* is the spin polarization of the alkali-metal atoms.

Ref. 10 brings forth a new method to measure the spin polarization. Instead of far-detuned probe light, the near-resonant probe light with two specific frequencies is chosen. And the detected Faraday rotation angles can be two orders of magnitude larger than that using far-detuned light, indicating a much more sensitive approach to measure the spin polarization of optically pumped alkali-metal atoms.

However, when the probe beam is near resonant, the absorption can no longer be ignored. As the linearly polarized probe beam transmits through the optically pumped alkali metal vapor, the occupation numbers of the energy levels of the alkali metal atoms will change, which, in turn, leads to modifications of the polarization state of the probe beam. This paper focus on the elliptical polarization of the near-resonant linearly polarized probe light after it goes through the optically pumped alkali metal atoms. A detailed theoretical analysis is given under the hypothesis of spin temperature distribution. The ellipticity of transmitted probe light is derived theoretically and measured experimentally. The negative impact of elliptical polarization on Faraday rotation measurements is also analyzed. This work has a great significance in both guaranteeing the sensitivity and calibrating the error in measuring the spin polarization utilizing near-resonant Faraday rotation.

## Methods

Suppose linearly polarized light incident on optically polarized alkali vapor along z axis. The electric field **E** of light can be decomposed into two orthogonal components, the left-circularly polarized component and right-circularly polarized component. The orthonormal basis vectors are denoted by


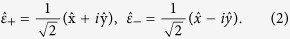


where 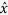 and 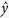, together with 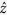, are the basis unit vectors in Cartesian coordinates.

Let us consider light linearly polarized along 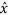. At the entrance face, the electric field could be written as


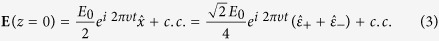


Here c.c. denotes the complex conjugate. After the linearly polarized light passes through the sample with a distance of *l*, left- and right-circularly polarized components experience different phase shifts, leading to optical rotation. In addition to the optical rotation of polarization plane, the incident light also experiences a difference in absorption between the two components (circular dichroism), which induces ellipticity in the output light. As a result, the linearly polarized light before the sample evolves into elliptical polarization after the sample^11^. The electric field of the transmitted light can be written as


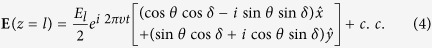


Here, *θ* is the polarization angle (azimuth) with respect to the x axis; *δ* represents the arctangent of the ratio of the minor to the major axis of the polarization ellipse, and 0 ≤ *δ* 0 < π/4. According to the spherical-basis unit vectors given in Eq. (2), we have


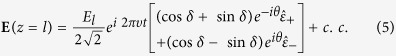


Generally, the high frequency terms are ignored and the transmitted intensity in terms of left- and right-circularly polarized components is given by


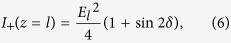



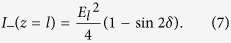


The absorption of near-resonant light propagating through the atomic vapor is corresponding to the imaginary parts of the refractive indices for the two circular components. The absorption cross section for left- and right-circularly polarized components of the linearly polarized light is symbolized by *σ*_+_ and *σ*_−_. The transmitted intensity satisfies


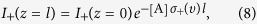



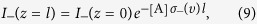


where [A] is the number density of the alkali metal atoms. As mentioned before, the light is linearly polarized before injecting on the optically polarized alkali vapor. We can easily obtain 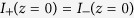 and


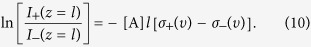


From Eq. (6), Eq. (7) and Eq. (10), the ellipticity of the output light satisfies


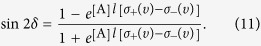


In general, the atomic frequency response depends on the natural broadening, pressure broadening, and Doppler broadening. The resulting lineshape of the atomic frequency response around the resonance frequency *υ*_0_ is the Voigt profile *V*(*ν* − *ν*_0_). Consider the hyperfine transition, the absorption cross section for the transition *F* → *F*′ is given by ref. 12


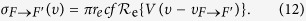


Here, 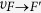 is the resonance frequency of the transition *F* → *F*′, *r*_*e*_ = 2.82 × 10^−15^ *m* is the classical electron radius, and *f* is the oscillator strength associated with the given resonance. 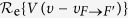 is the real part of 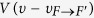.

The total photon absorption cross-section is


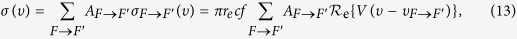


where 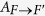 is the normalized relative strength for the transition *F* → *F*′^13^. According to Eq. (13), the absorption cross section for the left- and right-circularly polarized components are given as follows


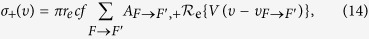



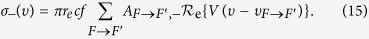


Here, 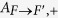 and 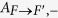 are the relative transition strengths of the left- and right-circularly polarized components, respectively.

Substituting Eq. (14) and Eq. (15) into Eq. (11), we can easily obtain


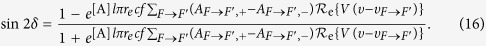


**Figure 1:**
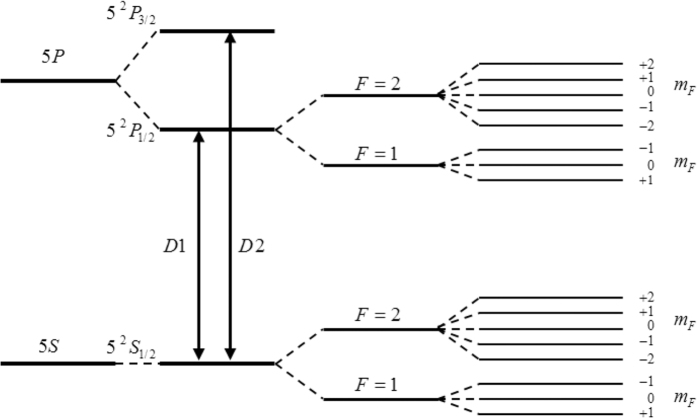
The energy level splitting of the ground state and the first excited state of ^87^*Rb* atoms.

If the left-circularly polarized light with a wavelength of 795 nm illuminates ^87^*Rb* vapor along z axis, the D1 transition of ^87^*Rb* is considered, as shown in Fig. 1. Due to quantum mechanical selection rules, the energy transitions are restricted to Δ*m*_*F*_ = 1. After a sufficient period of optical pumping by the left-circularly polarized pump light, the sublevel |*F* = 2, *m*_*F *_= 2〉 of the ground state 5^2^ *S*_1/2_ will thus be the most populated state. Under the durative pumping and relaxation procedure, as the system reaches the equilibrium state, the difference in the population among the other Zeeman energy levels expect |*F* = 2, *m*_*F*_ = 2〉 is tiny.

The difference values between the relative transition strengths 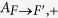 and 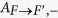 are given in Table 1^10^. 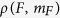 is the probability of the ^87^*Rb* atom being in the ground sublevel 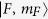.

Typically, when the alkali vapor density is high enough, rapid spin exchange collisions between two alkali-metal atoms lead to the spin-temperature distribution^14^. The relative populations 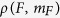 of the 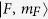 ground-state sublevels at an equilibrium state are well described by the with effective spin temperature *β*^15^:


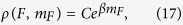


where 
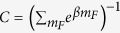
 is a normalization factor. The spin temperature is given by the spin polarization of ^87^*Rb* atoms along z axis *P*_*z*_


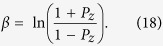


The spin polarization of ^87^*Rb* atoms can be modeled by solving rate equations^16^.

From Eq. (17, 18) and Table 1, Eq. (16) can be written as


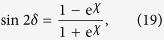


or


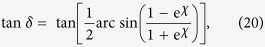


with


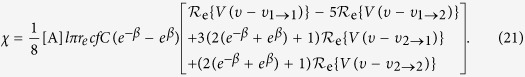


**Figure 2:**
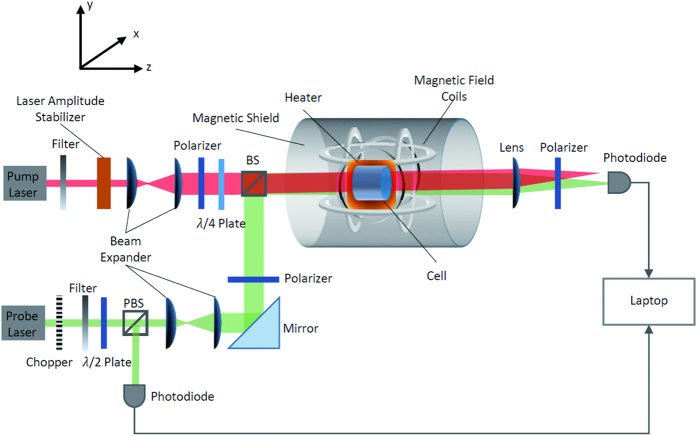
Experiment apparatus. BS: beam splitter, PBS: polarized beam splitter. A circularly polarized pump beam and a linearly polarized probe beam propagating along the z axis illuminate the vapor cell. A holding magnetic field *B*_0_ is imposed along the z axis. The ellipticity of the transmitted probe light is detected by rotating the orientation of the polarizer before the photodiode. The signal from the photodiode detector is collected by a DAQ system (not shown in Fig. 2) and transmitted to the laptop for calculation.

A typical experimental apparatus is shown in Fig. 2. We use a mixture of a droplet of ^87^*Rb* metal and 250 torr *N*_2_ of buffer gas for suppressing radiation trapping contained in an uncoated cylindrical glass cell which is 15 mm long and 10 mm in diameter. The pump beam and the probe beam go through the cylinder axis (z axis). The cell is placed in a non-magnetic oven located inside a five-layer cylindrical magnetic shields. Three pairs of Helmholtz coils compensate residual magnetic fields. After setting all three components of the external magnetic field to as near zero as possible, a holding magnetic field *B*_0_ of strength ~μT driven by a precision current source is oriented parallel to the pump beam to maintain the orientation of the polarized Rb atoms. The cell is heated to 350 K to 400 K by an electronic heater driven by AC currents at 210 kHz to achieve a desirable Rb number density ([Rb]~10^13^ cm^−3^), where a high SNR is guaranteed and a tremendous absorption of the probe laser light is avoided at the same time. A PID feedback controller adjusts the heating current so as to maintain constant temperature around the cell.

The circularly polarized pump beam resonant with the *F* = 2 → *F* = 1 transition of ^87^*Rb* D1 line propagates along the z direction. The frequency of the probe light is modulated so as to scanning around the *F* = 2 → *F* = 1 transition of ^87^*Rb* D1 line. A reference branch is inserted to the probe path for monitoring the frequency by a HighFinesse WSU wavelength meter. A 1/2 wave plate and PBS is added to tune the intensity distribution of the probe beam between the measurement branch and the reference branch. The pump beam and the probe beam originate from two DFB lasers and are expanded to illuminate the whole cell.

After the linearly polarized probe beam passing through the ^87^*Rb* vapor, difference in absorption between the left- and right-circularly polarized components of the light induces ellipticity in the output light. The ellipticity of the transmitted probe light is detected by rotating the orientation of the polarizer before the photodiode:


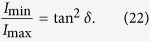


Here *I*_min_ and *I*_max_ are the minimal and maximal intensity around a circle respectively.

**Figure 3:**
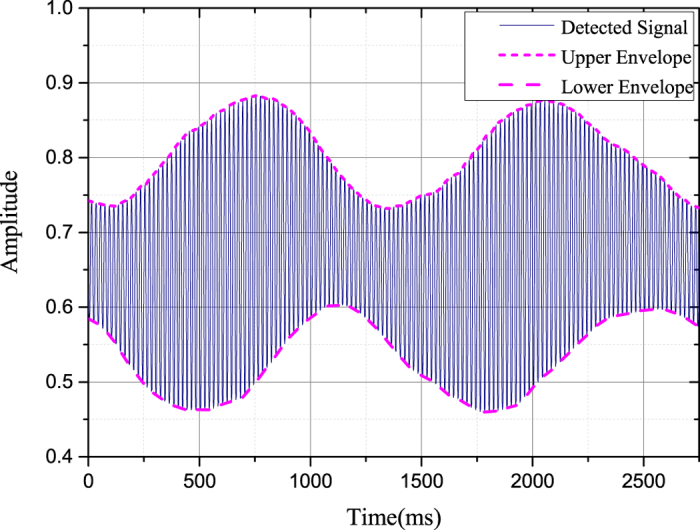
The signal detected by the photodiode after the cell. The chopper is utilized to eliminate the influence of the pump light. The difference value between the upper and lower envelope at the same time indicates the probe signal detected by the photodiode.

To eliminate the influence of the pump light, a chopper is inserted to shut off the probe beam periodically. The photodiode detects the transmitted probe light after the cell, as shown in Fig. 3. As we rotate the polarizer in front of the photodiode evenly, the upper and lower envelope in Fig. 3 oscillate like sine curves. The lower envelope origins from the scattering of the pump light and environmental influence. The difference value between the upper and lower envelope at the same time indicates the probe signal detected by the photodiode. The maximal and minimal difference stands for the maximal and minimal intensity in Eq. (22), respectively.

## Results and Discussion

Firstly, to make the results more persuasive, the ellipticity of the transmitted linearly probe light is measured at room temperature as reference. The pump light is shutoff and the holding magnetic field *B*_0_ is not applied. As we rotating the polarizer in front of the photodiode, the variation of the intensity are recorded. The reference ellipticity is approximately 0.0892 and mainly generated from the defect of the optical elements in the probe beam path.

**Figure 4:**
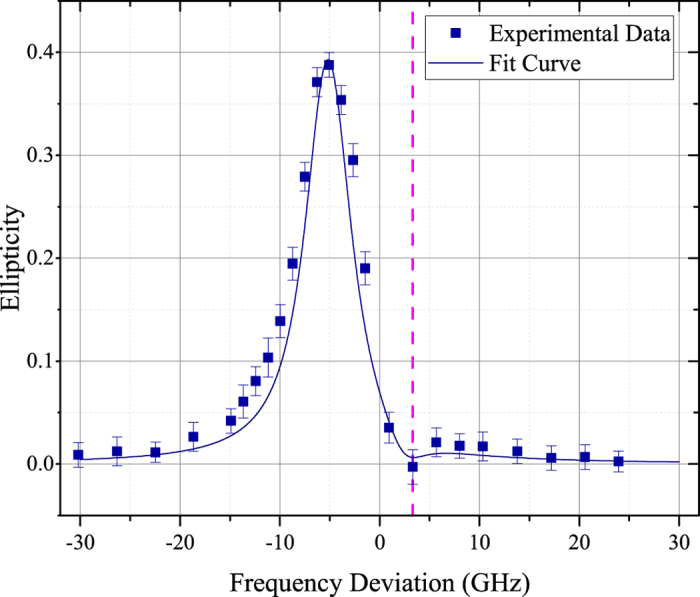
Experimental data (square dot) and the fitting curve (solid line) of the ellipticity 
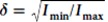
 as a function of frequency deviation from the *F* = 2 → *F* = 1 transition of ^87^*Rb* D1 line at 368 K. The intensity of the pump light is 80 mW. The Experimental data is revised according to the reference ellipticity measured at room temperature. The dashed line represents the specific frequency.

Next, the cell is heated to 368 K by the electronic heater. The pump power is adjusted to 150 mW and the probe light is modulated by the optical chopper at 50 Hz. When the chopper blades pass through and interrupt the probe beam path, we could get rid of the undesired output from the total signal detected by the photodiode. By rotating the polarizer in front of the photodiode, the ellipticity is derived from the minimal and maximal difference between the upper and lower envelope, as discussed above. As we scan the frequency of the probe light, the ellipticity 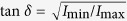 changes as a function of the frequency deviation from the *F* = 2 → *F* = 1 transition of ^87^*Rb* D1 line, as illustrated in Fig. 4. The frequency span covers the D1 (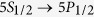) transition of ^87^*Rb*. The Experimental data is modified according to the reference ellipticity measured above. The fit curve is derived from Eq. (20) and Eq. (21). The experimental data and the fitting data are nearly identical. The ellipticity at large detuning is small and therefore can be ignored. However, ellipticity of near resonant reaches a high level, and the effect should be taken into account. Beyond that, a remarkable frequency arises in the ellipticity curve, represent by the dashed line in Fig. 4. The ellipticity curve of the transmitted probe light forms a small valley round this frequency. As shown in Fig. 4, the experimental lineshape is slightly broadened than theoretical simulation. This is because the light broadening effect^17^ is not taken into consideration in our model which could be compensated in further study.

**Figure 5:**
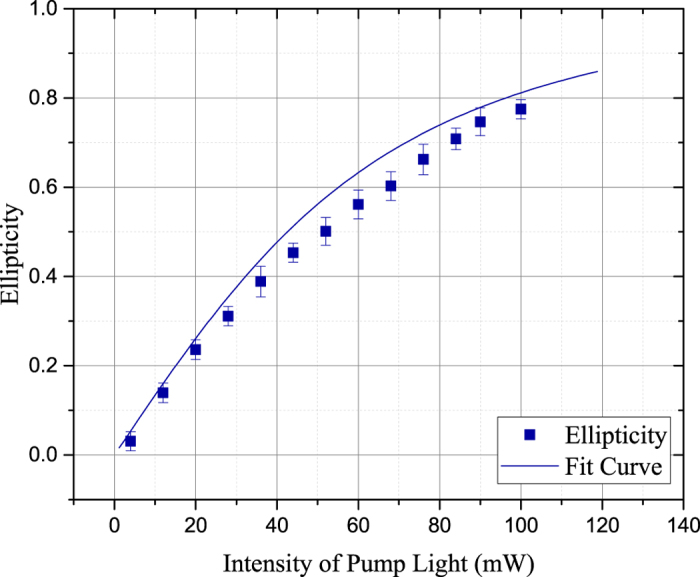
The experimentally measured and numerically calculated ellipticity of the transmitted probe light as a function of pump light intensity at 368 K. The probe light is locked at a specific frequency deviation 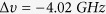. The Experimental data is revised according to the reference ellipticity measured at room temperature.

According to Eq. (20) and Eq. (21), the ellipticity is related to the polarization of ^87^*Rb* atoms in the vapor cell. Figure 5 illustrates the influence of the pump light intensity at 368 K. The probe light is locked at a specific frequency deviation 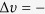
4.02 GHz. Our model fitted the data better if we use the pump intensity half what we measured, indicating the power loss in the path or a current problem with the model. From the gauge values, the ellipticity increase with the pump intensity. In other words, the polarization of ^87^*Rb* atoms will aggravate the elliptical polarization of the transmitted probe light.

**Figure 6:**
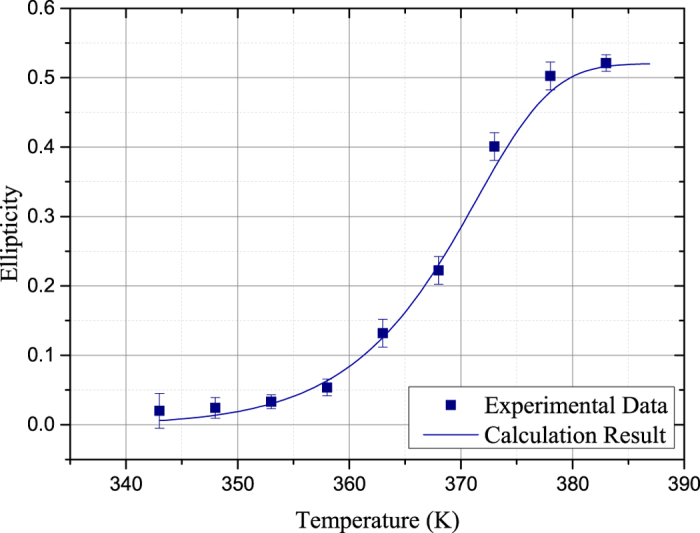
The experimentally measured and numerically calculated ellipticity of the transmitted probe light as a function of temperature in the vapor cell. The probe light is locked at a specific frequency deviation 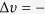
4.38 GHz. The Experimental data is revised according to the reference ellipticity measured at room temperature.

The ellipticity of the transmitted probe light as a function of temperature in the vapor cell is shown in Fig. 6. We lock the probe light at a constant frequency deviation 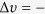
4.38 GHz. The ellipticity is obtained using the same strategy. Obviously, there is a positive correlation between the ellipticity and the temperature in a wide range. And this is mainly attributed to the elevated number density of Rb atoms as the cell gets warmer.

**Figure 7:**
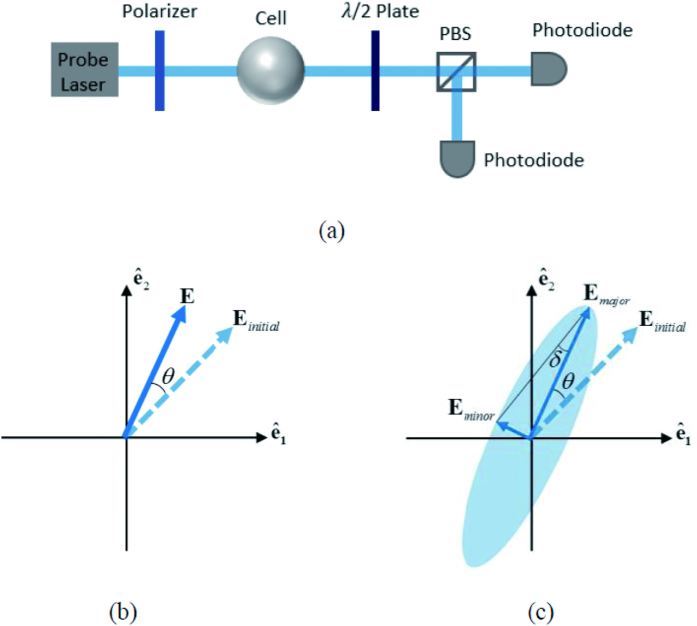
(**a**) A common apparatus detecting the optical rotation of the probe beam. (**b**) The ideal case: the linear polarization of the probe light is conserved after it pass through the alkali metal vapor cell; (**c**) the real case: the polarization state changes from linearly to elliptically.

As discussed above, ellipticity of near resonant probe light can’t be neglected, and should be taken into consideration in optical rotation measurements. A common method detecting the optical rotation of the probe beam is illustrated in Fig. 7(a). After passing through the cell, the probe beam reaches a polarized beam splitter set at 45° to the initial polarization. In the ideal case (Fig. 7(b)), the transmitted probe maintains the linear polarization state. Accordingly, the Faraday rotation angle *θ* can be deduced from the individual intensities:


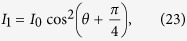



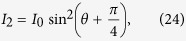


where *I*_0_ = *I*_1_ + *I*_2_. For small rotations, the rotation angle can be extracted from the difference between *I*_1_ and *I*_2_:


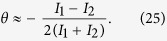


However, as discussed above, given the near-resonant probe light, the outcoming is no longer linear, shown in Fig. 7(c). The ellipticity of the light attaches an error on the acquired Faraday rotation angle *θ*. Under these conditions, the detected intensities satisfy


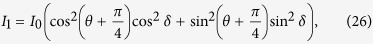



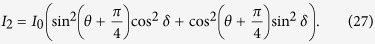


According to Eq. (25),


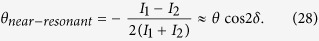


As a consequence, when the near-resonant light is applied to detecting the Faraday rotation, the accompanied elliptically polarization will reduce the result by a factor of cos 2*δ*. Referring to the theoretical and experimental studies above, the scale factor cos 2*δ* is, in the main, positively related to the frequency deviation of the probe light, the polarization and number density of alkali metal atoms.

In conclusion, we have demonstrated the existence of the elliptical polarization of the near-resonant linearly polarized probe light when it transmits optical pumped alkali metal vapor. Experimental results are in good agreement with the theoretical predictions. The elliptical polarization is proved to reduce the result of Faraday rotation by a factor of cos 2*δ*, which is positively related to the frequency deviation of the probe light, the polarization and number density of alkali metal atoms. Thus, work of this paper can be used to eliminate the error and improve the sensitivity of magnetic microscopic measurements. In addition, elliptical polarization of the transmitted near-resonant linearly polarized probe light also provides psotential in spin polarization measurements, and further research is required to demonstrate this application.

## Additional Information

**How to cite this article:** Li, Y. *et al*. Elliptical polarization of near-resonant linearly polarized probe light in optically pumped alkali metal vapor. *Sci. Rep.* **7**, 43066; doi: 10.1038/srep43066 (2017).

**Publisher's note:** Springer Nature remains neutral with regard to jurisdictional claims in published maps and institutional affiliations.

## Figures and Tables

**Table 1:** The difference value 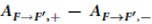 for the ^87^ *Rb* D1 transition.

*F* → *F*′	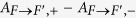
1 → 1	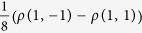
1 → 2	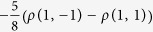
2 → 1	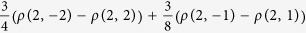
2 → 2